# Improving future care: the role of oncofertility education in medical curricula in Germany. Results of a nationwide survey of 346 medical students

**DOI:** 10.1007/s00404-026-08425-z

**Published:** 2026-04-29

**Authors:** Judith Altmann, Desislava Dimitrova, Elena Stark, Nadine Einsiedel, Lea Heistermann, Jalid Sehouli

**Affiliations:** Department of Gynecology with Center for Oncological Surgery, Charité - University Hospital Berlin, Charitéplatz 1, 10117 Berlin, DE Germany

**Keywords:** Oncofertility, Fertility preservation, Fertility sparing, Fertility assessment, Medical education, Academic training

## Abstract

**Objective:**

To study the existing knowledge, interests, and attitudes of medical students regarding fertility preservation measures.

**Design:**

A multi-center nationwide survey was conducted among medical students across Germany. The survey consisted of 15 multiple-choice questions and six questions asking participants their level of agreement of a statement.

**Subjects:**

346 medical students in Germany.

**Main outcome measures:**

A multiple-choice questionnaire was used to evaluate the existing knowledge, interest and attitudes of medical students regarding fertility preservation measures.

**Results:**

Regarding the assessment of ovarian reserve, Anti-Müllerian hormone testing was known by 38.8% of students, while only 15.9% were familiar with the antral follicle count method. The most widely recognized fertility preservation (FP) method was oocyte cryopreservation (91.0%), followed by ovarian tissue cryopreservation (45.1%), ovarian transposition surgery prior to pelvic radiation (38.2%), and the use of gonadotropin-releasing hormone analogs for ovarian protection during chemotherapy (28.6%). Regarding reproductive technologies, such as IVF, ICSI, or hormonal stimulation, 87.5% of students expressed a positive opinion. Oocyte donation was perceived positively by 77.6%. The acceptance for uterus transplantation was high among participants with 61.4%. The acceptance for surrogacy was lower with 53.2% of respondents. Social freezing was positively received by most respondents (72.7%). 80.0% of respondents expressed interest in expanding their knowledge of fertility and cancer, and 65.9% desired additional training.

**Conclusions:**

To ensure high-quality fertility preservation counseling and the implementation of necessary FP measures for all cancer patients of childbearing age, it is crucial that future physicians are familiar with methods for assessing ovarian reserve and fertility preservation techniques. Strengthening oncofertility training in medical education will enhance future physicians’ ability to provide informed counseling and timely referrals, ultimately improving patient care and quality of life. This study highlights the urgent need to systematically integrate fertility preservation and oncofertility into the curriculum of medical schools in Germany.

**Supplementary Information:**

The online version contains supplementary material available at 10.1007/s00404-026-08425-z.

## What does this study add to the clinical work?

**Table:** 

This is the first study evaluating existing knowledge, interest and attitudes of medical students regarding fertility preservation measures in Germany.
This study highlights the urgent need to systematically integrate fertility preservation and oncofertility into the curriculum of medical school in Germany.

## Introduction

Infertility and premature ovarian insufficiency as a result of cancer treatment significantly increase the burden that young cancer patients must carry [1, 2], leading to unintended childlessness, menopausal symptoms, osteoporosis, sarcopenia, and an increased cardiovascular risk.

All cancer patients of reproductive age, regardless of the type and stage of the disease, should receive comprehensive oncofertility counseling as early as possible in the treatment planning process, as stated in the ASCO and ESMO guidelines [3, 4]. In Germany, as of July 2021, cancer patients are even entitled to statutory health insurance coverage for fertility-preserving treatments [5]. Despite the existence of this and several other American or European guidelines [3, 4, 6, 7] studies highlight that many patients still do not receive adequate counseling or referrals to reproductive medicine specialists [1, 8].

When asked about the reasons for not offering fertility preservation counseling to gynecological cancer patients, doctors cite a lack of knowledge, insufficient prioritization, and time constraints [8]. Medical students, as tomorrow’s healthcare providers, are the key to improving fertility preservation counseling in the future, making their education on the topic critical for enhancing care quality.

The educational framework of medical curricula in Germany is based on the principles of competency-based medical education. Nationwide learning objectives are defined in the National Competency-Based Learning Objectives Framework Medicine (NKLM), which serves as the central reference for competency development in undergraduate medical training.

The primary objective of the present study is to identify knowledge gaps of medical students in fertility preservation techniques and counseling for cancer patients. As a secondary objective, medical students’ attitudes toward reproductive technologies and alternative pathways to parenthood are explored.

## Methods

Prior to commencing the study, approval was obtained from the Ethics Committee of Charité. A multi-center nationwide survey was conducted among medical students across Germany. All participants provided written informed consent to participate in the study.

The survey was developed based on guidelines on fertility preservation in females [3, 4, 6] as well as on expert consensus since no validated instrument was available to address our research questions. Content validity was assessed by an expert panel consisting of specialists in reproductive medicine, gynecology, and medical education. Furthermore, a pilot phase involving interviews with 10 medical students was conducted to confirm the comprehensibility and feasibility of the questionnaire.

The survey consisted of 15 multiple-choice questions and six questions asking participants their level of agreement with the statement on a scale from 0 (= completely disagree) to 10 (completely agree) in German. In our analysis we considered values under five as disagreement, five as neutral and above five as agreement. Knowledge questions were multiple choice.

The questionnaire gathered information on respondents’ demographics, such as gender, personal expertise, and knowledge related to fertility preservation counseling, and whether this topic had already been covered in university classes. Knowledge of different techniques and measures of fertility preservation was tested. The last section asked participants about their personal opinions regarding fertility preservation measures, including procedures not yet legalized or established in Germany (see supplementary material).

The survey was distributed online from 03/2024 to 08/2024 via mailing lists and student associations of medical universities across Germany. The exact response rate cannot be determined due to the nature of the survey distribution. For context, 117,916 medical students were officially registered in Germany in 2024 [9].

Categorical data were presented as percentages. Percentages were calculated on the basis of the total number of responses for each question individually and rounded to one decimal place.

For conducting the online survey, we used the software REDCap. Figures were created using the R package ggplot2 [10].

Exploratory statistical analysis was performed using IBM SPSS Statistics (version 29.0.2.0.(20)). Odds ratios (ORs) with 95% confidence intervals (CIs) were calculated to quantify associations.

Analyses were conducted using univariate tests.

To evaluate potential associations between the students’ academic progress and the extent of their knowledge, a bivariate analysis was performed between the knowledge of AMH as a marker of fertility and the study progress (grouped into 1st–4th semester, 5th–8th semester and 9th–13th semester).

To assess the influence of practical experience in the field of gynecology on students’ knowledge, a bivariate analysis was conducted between knowledge of any method used to assess ovarian reserve and the variable of having practical experience in the field of gynecology. Knowing any measurement of assessing ovarian reserve included knowledge of AMH levels, antral follicle count (AFC) and measurement of luteinizing hormone (LH), follicle-stimulating hormone (FSH) and estrogen.

## Results

### Demographic characteristics of respondents

A total of 346 medical students answered the online survey. Students were distributed as follows across their medical studies: 20.8% in the 1st–4th semester, 47.7% in the 5th–8th semester, and 31.5% in the 9th–13th semester of their studies. 43.4% of students had already studied gynecology as part of their curriculum, and 43.6% had acquired practical experience in this field. 36.1% of students stated that they had attended a seminar on “fertility preservation in cancer patients” in university.

80.3% of the respondents identified as female, 18.2% as male, 1.2% as non-binary, and 0.3% chose not to respond to this question. In terms of parental status, a significant majority of participants, specifically 92.5%, reported not having children of their own. Furthermore, 3.5% of the participants indicated that they had utilized the services of a reproductive medicine specialist at some point.

When asked to rate the importance of having biological children on a scale from 0 to 10, 74.4% of participants considered it to be somewhat to very important to them personally.

For a detailed breakdown of demographic characteristics, refer to Table 1.

**Table 1:** Characteristics of respondents

%		
Total number *n* = 346		
Semester		
1.–4. semester	72	20.8%
5.–8. semester	165	47.7%
9.–13. semester	109	31.5%
No answer	0	0
Did you already cover gynecology as part of your academic curriculum?		
Yes	150	43.4%
No	193	55.8%
No answer	3	0.9%
Do you have practical experience in gynecology?		
Yes	151	43.6%
No	194	56.1%
No answer	1	0.3%
Gender identity		
Female	278	80.3%
Male	63	18.2%
Diverse	4	1.2%
No answer	1	0.3%
Did your studies include any course or seminar on fertility preservation and cancer?		
Yes	125	36.1%
No	205	59.2%
No answer	16	4.6%
Do you have children?		
Yes	26	7.5%
No	319	92.5%
No answer	0	0
Missing answer	1	0.3%
Have you personally utilized options of fertility preservation?		
Yes	12	3.5%
No	330	95.7%
No answer	3	0.9%
Missing answer	1	0.3%

### Need for training

When asked about their interest in “fertility and cancer,” 80.0% of participants expressed a desire to gain more knowledge in reproductive medicine, while 15.9% responded with “no interest,” and 4.1% did not provide an answer. 65.9% of participants expressed interest in further classes and training on “fertility and cancer,” while 17.1% responded with “no,” and 17.1% did not provide an answer. 35.1% preferred further training on “fertility and cancer” through a clinical rotation at a specialized center, 29.4% favored in-person training, and 22.4% preferred an online course. Even among medical students who had already encountered fertility-related topics in their curriculum, there was a strong demand for additional education and training opportunities.

Only 14.5% of students stated that “fertility and cancer” was adequately covered during their studies, while 44.1% felt the topic was not sufficiently addressed, and 41.4% did not provide an answer (see Table 2).

**Table 2:** Knowledge and attitudes of medical students regarding fertility preservation

	*n*	%
Would you personally like to have more expertise in reproductive medicine?		
Yes	276	79.8%
No	55	15.9%
No answer	14	4.1%
Missing data	1	0.3%
Is the topic of fertility and cancer sufficiently represented in your studies?		
Yes	50	14.5%
No	152	43.9%
No answer	143	41.3%
Missing data	1	0.3%
Do you wish for more extensive training programs?		
Yes	228	65.9%
No	59	17.1%
No answer	59	17.1%
If so, which format would you prefer?		
Face-to-face training	67	29.4%
Webinar	51	22.4%
Crash course over several days	28	12.3%
Rotation to a specialized clinic/practice	80	35.1%
No answer	1	0.4%
Other	1	0.4%
Missing data	118	34.1%
Which methods for assessing ovarian reserve do you know? (multiple answers)		
AMH measurement	134	38.8%
Antral follicle count	55	15.9%
LH, FSH, estrogen measurements	194	56.2%
None	100	29.0%
Missing data	1	0.3%
Which of the following measures for preserving fertility do you know? (multiple answers)		
Cryopreservation of ovarian tissue	156	45.1%
Cryopreservation of oocytes	315	91.0%
Gnrh analogs to protect the ovaries during chemotherapy	99	28.6%
Surgical relocation of the ovaries prior to radiation	132	38.2%
None	13	3.8%

### Knowledge regarding fertility preservation

**Fig. 1:**
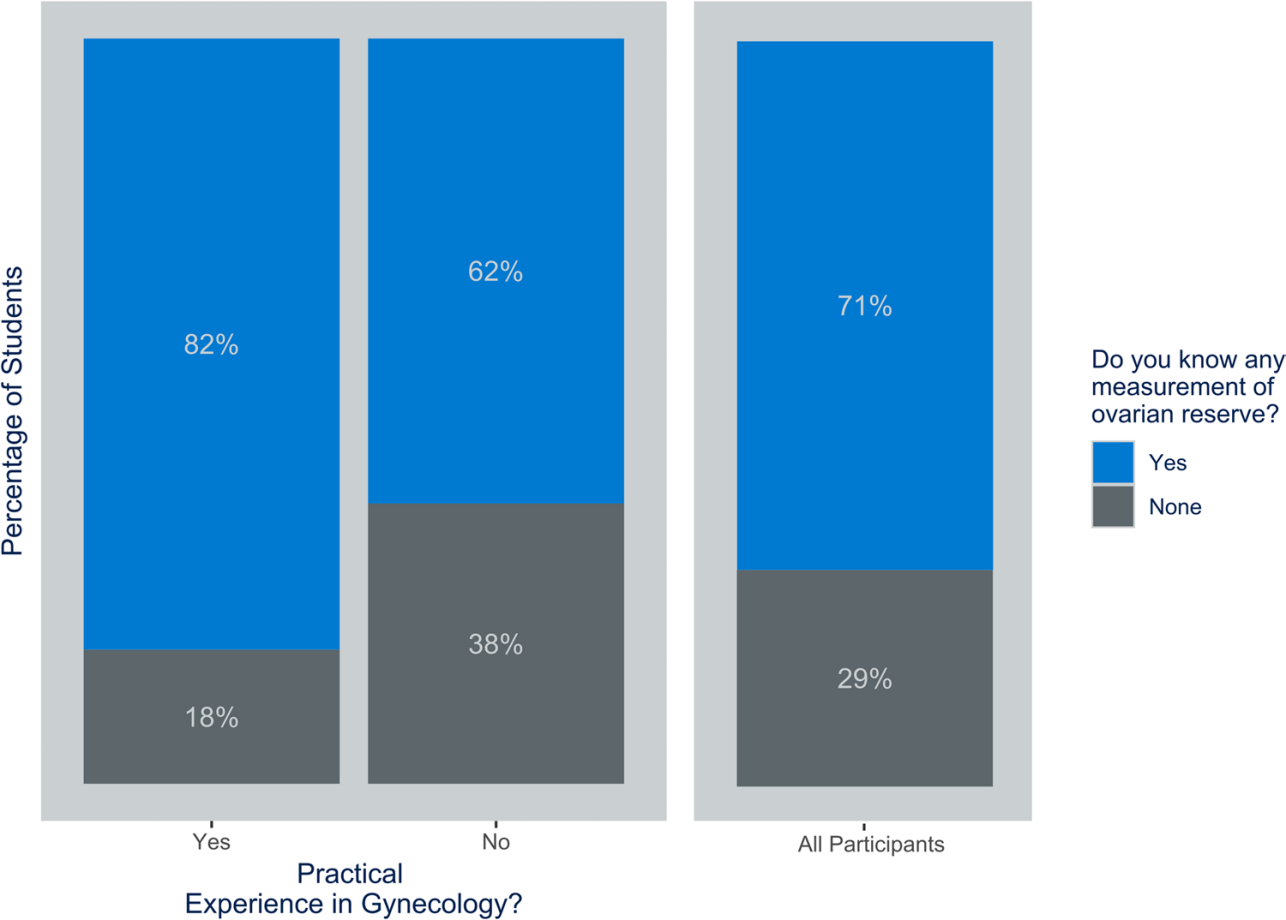
Knowledge of any methods of assessing ovarian reserve in relation to practical experience

When asked about their knowledge of methods to assess ovarian reserve in women, 29% of students were unaware of any (Fig. 1).

**Fig. 2:**
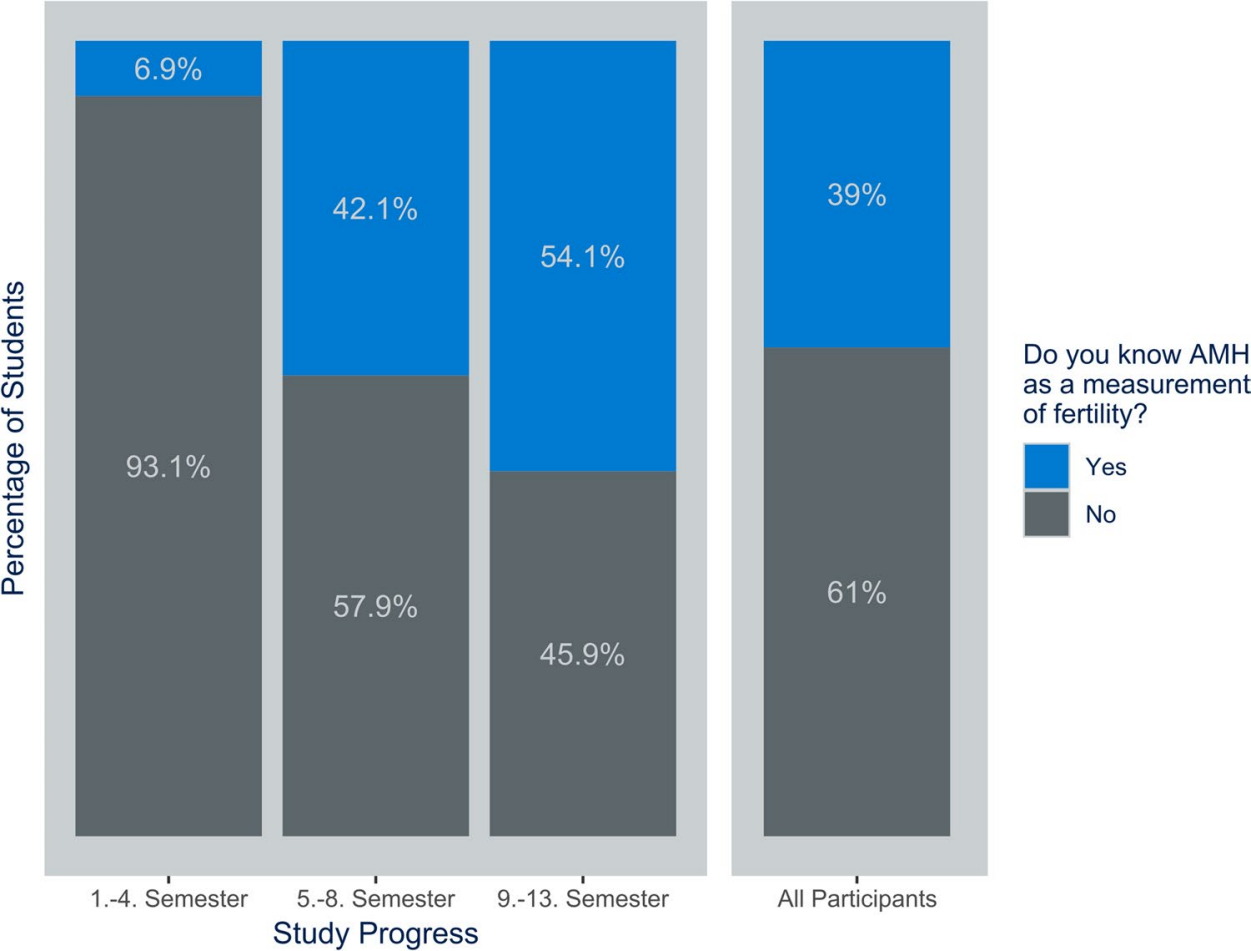
Knowledge of AMH (Anti-Mullerian Hormone) as a measurement of ovarian reserve in relation to study progress

The most recognized approach was hormonal measurement, including luteinizing hormone (LH), follicle-stimulating hormone (FSH), and estrogen (56.2%). Anti-Müllerian hormone (AMH) testing was known by 38.8% of students (see Fig. 2), while only 15.9% were familiar with the antral follicle count (AFC) method.

Students with practical experience in the field of gynecology (e.g. through internships) were significantly more likely to know of any measurement of ovarian reserve than those without (OR = 1.960; CI = 1.261–3.046) (Fig. 1).

Furthermore, while in the first four semesters only 6.9% of students knew AMH as a measurement of fertility, this proportion increased to 42.1% among students in the fifth to eighth semesters and to 54.1% among students in the ninth to thirteenth semesters (Fig. 2). This increase throughout the semesters was statistically significant with students in the intermediate stage of their studies being nearly ten times more likely (OR = 9.733; CI = 3.726–25.420) and in the final stage more than fifteen times more likely (OR = 15.812; CI = 5.913–42.284) to know AMH as a marker of fertility compared to students in the initial stage. Other methods for assessing ovarian reserve and students’ knowledge of various fertility preservation techniques were also analyzed. No significant differences across semesters were observed. The only statistically significant finding was that students in higher semesters were less likely to report being unfamiliar with any of the methods.

The most widely recognized fertility preservation (FP) method was oocyte cryopreservation, acknowledged by 91.0% of participants, followed by ovarian tissue cryopreservation (45.1%), ovarian transposition surgery prior to pelvic radiation (38.2%), and the use of gonadotropin-releasing hormone (GnRH) analogues for ovarian protection during chemotherapy (28.6%). A small percentage (3.8%) of students were unaware of any of these methods. Notably, students were only asked whether they were aware of these methods, without being required to provide detailed comprehension.

Regarding age limits for offering fertility preservation, 1.4% of participants believed FP should be available to women only up to the age of 30, 15.9% up to 35, 38.7% up to 40, and 38.4% up to 45. Meanwhile, 5.5% did not provide an answer.

Most respondents somewhat to fully agreed that fertility-preserving measures should be offered to patients with a very good disease prognosis (89.0%). Additionally, 94.5% somewhat to fully supported offering these measures to patients with a risk of amenorrhea greater than 20%, and 83.8% supported offering these measures to those who had not yet had children. Furthermore, 86.1% and 78.9% endorsed fertility preservation only when the efficacy of oncological therapy was not compromised or delayed, respectively.

When asked to assess the safety of assisted reproductive technologies, such as in vitro fertilization (IVF), intracytoplasmic sperm injection (ICSI), or hormonal stimulation in patients with a history of hormone-receptor-positive tumors, respondents rated these methods as very to somewhat unsafe (51.2%), neutral (19.8%), and somewhat to very safe (29.1%).

Similarly, 48.4% of participants viewed the prospect of a future pregnancy in a patient with a history of a hormone-receptor-positive tumor as very to somewhat unsafe for the oncological course of the disease, while 19.0% remained neutral and 32.7% considered it somewhat to very safe.

Lastly, 97.7% of participants believed that a patient’s wish to preserve fertility was somewhat to very important.

### Attitudes of medical students concerning fertility preservation

Only 6.4% of respondents viewed reproductive technologies, such as IVF, ICSI, or hormonal stimulation negatively, while 6.1% remained neutral and 87.5% expressed a positive opinion.

Regarding the cryopreservation of ovarian tissue, 5.2% regarded this method negatively, 9.3% remained neutral, and 85.5% viewed it positively.

Oocyte donation was perceived negatively by 12.8% of respondents, neutrally by 9.6%, and positively by the remaining 77.6%.

The acceptance for uterus transplantation was high among the participants with 61.4% responding positively (21.2% responding negatively, 17.4% responding neutrally). The acceptance for surrogacy was lower but more than half of students had a positive attitude (31.7% responding negatively, 15.1% responding neutrally, and 53.2% responding positively).

Social freezing was positively received by most respondents (72.7%), while 9.0% remained neutral and 18.3% viewed it negatively.

When asked about the legalization of these procedures, 67.5% supported the legalization of oocyte donation, 24.6% were unsure, and 7.8% opposed it.

Regarding uterus transplantation, 45.2% approved, 46.4% were uncertain, and 8.4% disapproved.

Surrogacy legalization was opposed by 22.6% of respondents, while 42.3% were undecided and 35.1% supported it.

## Discussion

This is the first study aimed at investigating the awareness, knowledge, and attitudes of German medical students regarding fertility preservation and fertility preservation counseling, as well as identifying potential gaps in their academic training. The results from our survey suggest that knowledge in the area of fertility preservation remains heterogeneous. While 91.0% of participants were familiar with oocyte cryopreservation, fewer than half were aware of ovarian tissue cryopreservation, ovarian transposition surgery prior to pelvic radiation, or the use of GnRH analogues for ovarian protection during chemotherapy. This likely reflects the fact that oocyte cryopreservation has gained broad recognition in the general population and is increasingly sought after in the context of “social freezing,” which allows women to preserve fertility for later use due to the absence of a partner, career considerations, or other social factors. AMH measurement as a method for assessing ovarian reserve was known to less than half of the participants, and only 15.9% were familiar with the AFC method. 29.0% of participants were unaware of any methods for measuring ovarian reserve. The most commonly reported answer was LH, FSH, and estrogen measurement, which, however, is a misconception, as these do not directly indicate ovarian reserve. Notably, students were not asked to provide any detailed knowledge; they were only if they had heard of these methods. These findings on knowledge of fertility preservation methods and ovarian reserve assessment reveal significant clinical knowledge gaps that should be addressed in the medical curriculum to optimize patient care.

As their academic training progressed, students became noticeably more knowledgeable about fertility preservation topics, likely reflecting both academic coursework and practical experience within the field of gynecology.

Regarding age limits, more than a third of respondents would offer fertility preservation measures to patients up to 45 years of age. This could be attributed to an overestimation of female fertility at that age, but it may also reflect a changing demographic trend, with women becoming mothers at increasingly older ages and social freezing emerging as an option for delayed childbearing. However, several studies show that even medical students tend to overestimate female fertility and underestimate age-related fertility decline [11, 12].

On a broader societal level, this is concerning, as education at both school and university levels primarily focuses on preventing unwanted pregnancies while rarely addressing the age-related risks of infertility, despite the fact that childbearing age continues to rise in Western countries [13]. Knowledge of age-related infertility should ideally be integrated early into the medical curriculum and also included in high school education to enhance public awareness of this issue.

Another important aspect is the safety of reproductive measures and pregnancies in patients who have undergone treatment for hormone receptor-positive tumors. Within our cohort, a significant level of uncertainty was observed on this matter, with only a third of participants considering it safe from an oncological perspective. This uncertainty also persists within the broader medical community, which is particularly concerning, as it may limit young patients’ access to fertility preservation and opportunities for future pregnancy. Studies suggest that reproductive measures and pregnancies are safe and do not negatively affect tumor prognosis [14], recurrence, mortality or event-free survival [15] in young breast cancer patients. Whereas robust evidence is available for patients with breast cancer, further research into other tumor types is urgently needed. Evidence-based safety data regarding hormonal stimulation and pregnancy after cancer should be included in medical curricula to address this issue.

While the first part of the survey was dedicated to identifying knowledge gaps in fertility preservation, the second part examined personal attitudes toward various techniques and methods. Our survey demonstrated an overwhelmingly positive attitude toward reproductive measures, such as IVF, ICSI, and social freezing. This may be related to the fact that female medical students often face the challenge of balancing family planning with career advancement, given that medical education and specialist training in Germany take an average of 11 years to complete and most of this survey’s respondents were female. As a result, medical students and doctors tend to delay childbearing in order to meet the demands of this time-intensive medical training and the working conditions in hospitals. This is a global trend [16], to the extent that infertility is more common among U.S. doctors than in the general population [16]. Studies conducted among U.S. medical students revealed that the majority would consider freezing their oocytes if their employer covered the costs [17].

### Non-legal options in Germany

Until today, Germany’s legislation remains more conservative than that of many other European and Western countries regarding reproductive technologies [18]. Oocyte donation is still prohibited under German Law [18], while it is legal in countries, such as France, Spain, Italy, Denmark, and the Czech Republic [19]. Similarly, surrogacy, which is legal under certain conditions in Denmark and the United Kingdom and fully permitted in Ukraine, remains illegal in Germany [18]. For women unable to carry a pregnancy due to anatomical anomalies, cancer surgery, or other factors, uterus transplantation offers a potential solution. Since the first successful deceased donor transplantation in 2011 and the first live donor birth in 2014, over 30 procedures have been performed worldwide. However, this remains an experimental approach unavailable to most patients [20]. As a result, reproductive options for young cancer survivors facing infertility due to cancer treatment are significantly limited.

Our study indicates a more progressive stance on these issues among medical students compared to the general German population. While a clear majority of our cohort supported oocyte donation, a 2009 survey among the German population found that only ~ 51% approved of oocyte donation, with ~ 36% supporting it for medical reasons and just ~ 13% favoring general approval [21]. However, given that this survey was conducted almost 20 years ago, public attitudes may have shifted, although the issue remains controversial. This societal trend is also reflected in a statement by the German National Academy of Sciences Leopoldina, advocating for the legalization of oocyte donation, as the Embryo Protection Law “no longer reflects societal changes and the diversity of modern family structures” [22].

Meanwhile, opinions on surrogacy remained divided within our cohort, with only a third supporting its legalization. Similarly, approval rates for uterus transplantation were only around 45%. For comparison, 45% of healthcare providers and 78% of the general population in the USA approve of uterus transplantation [23, 24]. When asked whether oocyte donation, surrogacy and uterus transplantation should be legalized, a significant proportion of students remained undecided: ~ 25%, ~ 42% and ~ 46% respectively. However, our survey did not explore whether this uncertainty regarding legal endorsement stemmed from ethical conflicts, professional or scientific considerations or a lack of engagement with the topic. In light of the substantial societal relevance of these ethical and legal issues, medical curricula should not focus solely on knowledge acquisition but also foster ethical and legal discourse through case-based and problem-based learning in complex clinical scenarios, enabling students to develop informed perspectives and engage in societal debates.

### Interest in further courses and training

The vast majority of students expressed a high interest in expanding their knowledge. Students believed that there is a gap in their academic education on “fertility and cancer” and demanded further courses and training preferably in the form of a clinical rotation, in-person teaching or an online course, in this order.

80.0% of respondents expressed interest in expanding their knowledge of fertility and cancer, and 65.9% desired additional training. While students show interest in the topic throughout all semesters of medical school and knowledge rises significantly with the course of medical school, even in higher semesters general knowledge of fertility preservation techniques and assessment of ovarian reserve remains limited.

A strong desire for expanding their knowledge in fertility preservation has also been shown by studies in Hong Kong [12] and in the USA [25].

Based on students’ preferences and the identified knowledge gaps, we recommend strengthening the coverage of fertility preservation within the National Learning Objectives Catalog for Medical Education in Germany. Additional educational opportunities could be provided through clinical rotations in gynecology, reproductive medicine, and oncology, as well as through interdisciplinary seminars and digital learning modules. Understanding gonadotoxic effects and fertility preservation options is essential for all physicians, regardless of their medical subspecialty, and therefore should be an integral part of the medical curriculum. Almost all medical subspecialties, not only gynecologic oncology, urological oncology, hematology/oncology, radiology, and radiation oncology, frequently encounter gonadotoxic treatments and fertility-related decisions. Hence, specialists should ensure timely referral of patients to reproductive medicine experts and facilitate multidisciplinary discussions of treatment strategies, taking into account the gonadotoxic potential of therapies.

The emphasis on education is crucial, as the decision to offer counseling on FP significantly impacts not only tumor prognosis, but also the incidence of premature ovarian insufficiency, menopausal symptoms, osteoporosis, sarcopenia, and cardiovascular risk profiles. Moreover, it contributes to lower levels of psychological distress among patients [26–28]. Even if patients ultimately decide against fertility preservation after receiving counseling, their satisfaction with and adherence to oncological treatment tend to improve [25, 26]. Active involvement in decision-making and feeling well-informed about their options also contribute to an enhanced quality of life for patients [26–28].

With these results, we hope to contribute to the scientific foundation for expanding and refining the medical curriculum in Germany.

### Limitations of the study

The survey only represents the situation in Germany, limiting the generalizability of our findings. Moreover, the variability in medical curricula across Germany may have influenced participants’ knowledge and exposure to fertility preservation. Noting the high proportion of female respondents in our study (80%) compared to the 65% reported among medical students in Germany [8], as well as differences in the demographics of our participants and the broader population of medical students in Germany, we acknowledge that our sample may not fully represent medical students overall. The overrepresentation of female participants may be related to differing levels of interest among female, male, and diverse medical students, potentially resulting in non-response bias. The survey relied on self-reported data, which may introduce response bias. Furthermore, due to the voluntary nature of participation in our online survey, the study may be subject to self-selection bias. These contextual factors should be considered when interpreting our findings.

## Conclusion

In conclusion, this study highlights the urgent need to systematically integrate fertility preservation and oncofertility into the curriculum of medical schools in Germany, taking into consideration the limited knowledge but stark interest medical students displayed.

To ensure high-quality fertility preservation counseling and the implementation of necessary FP measures for all cancer patients of childbearing age, it is crucial that future physicians are familiar with methods for assessing ovarian reserve and fertility preservation techniques. Strengthening oncofertility training in medical education will enhance future physicians’ ability to provide informed counseling and timely referrals, ultimately improving patient care and quality of life.

## Supplementary Information

Below is the link to the electronic supplementary material.

Supplementary file1 (DOCX 24 KB)

## Data Availability

Data will be shared upon request.

## Declarations

### Attestation statements

All authors confi rm that the data are accurate, the study was conducted in accordance withethical standards, and the manuscript has been reviewed and approved by all authors.

### Capsule

This is the first study evaluating existing knowledge, interest and attitudes of medical students regarding fertility preservation measures in Germany.
