# Supplementary material for: Improving future care: the role of oncofertility education in medical curricula in Germany. Results of a nationwide survey of 346 medical students
Source: Arch Gynecol Obstet. 2026 Apr 29;313(1):187. doi: 10.1007/s00404-026-08425-z (PMC13128717; doi:10.1007/s00404-026-08425-z)
Supplement: Supplementary file 1 — Supplementary file1 (DOCX 24 KB) [file 404_2026_8425_MOESM1_ESM.docx]

**Supplementary materials**

**Questionnaire**

**1. In which semester are you currently enrolled?**

- 1st–4th semester
- 5th–8th semester
- 9th–13th semester
- No answer

**2. Have you already completed the course in gynecology during your studies?**

- Yes
- No
- No answer

**3. Have you gained any practical experience in gynecology?**

- Yes
- No
- No answer

**4. What is your gender?**

- Female
- Male
- Diverse / Non-binary
- No answer

**5. Have you attended any lecture, seminar, or course on fertility and cancer during your studies?**

- Yes
- No
- No answer

**6. Would you personally like to gain more expertise in reproductive medicine?**

- Yes
- No
- No answer

**7. Do you feel that fertility and cancer are sufficiently covered in your curriculum?**

- Yes
- No
- No answer

**8. Would you like additional education or training on this topic?**

- Yes
- No
- No answer

**8.1 If yes, which format would you prefer additional education or training?**

- In-person training
- Webinar
- Multi-day crash course
- Rotation at a specialized clinic/practice
- No answer
- Other: __________

**9. Which methods for assessing ovarian reserve are you familiar with?** (*Multiple answers possible*)

- AMH measurement
- Antral follicle count (AFC)
- LH, FSH, estrogen measurements
- None

**10. Which of the following fertility preservation measures are you familiar with?** (*Multiple answers possible*)

- Ovarian tissue cryopreservation
- Oocyte cryopreservation after hormonal stimulation
- GnRH analogues for ovarian protection during chemotherapy
- Surgical ovarian transposition prior to planned radiation
- None

**11. Which patients should be offered fertility preservation measures?**

Patients of the following ages:

- Up to 30 years
- Up to 35 years
- Up to 40 years
- Up to 45 years
- No answer

**12. To what extent do you agree that fertility preservation should be offered to patients in the following situations?**

*(scale from 0 to 10; 0 = strongly disagree, 10 = strongly agree)*

- In case of very good disease prognosis
- For patients with a >20% risk of amenorrhea (e.g., due to radiation or chemotherapy)
- For patients who do not yet have children
- If oncological treatment effectiveness is not compromised
- If no additional delay of oncological treatment occurs

**13. From an oncological standpoint, how confident are you in recommending ____________ for patients with hormonally sensitive tumors?**

*(scale from 0 to 10; 0 = very uncertain, 10 = very confident)*

- Fertility treatment (intracytoplasmic sperm injection [ICSI] and/or in vitro fertilization [IVF] or hormonal stimulation)
- Pregnancy

**14. Do you have children yourself?**

- Yes
- No
- No answer

**15. How important is it to you personally to have children?**

*(scale from 0 to 10; 0 = not important, 10 = very important)*

**16. How important, in your opinion, is it for patients with gynecological cancers to have…**

*(scale from 0 to 10; 0 = not important, 10 = very important)*

- …the opportunity to fulfill their desire to have children
- …their fertility preserved

**17. Have you personally taken any measures to preserve your fertility?**

- Yes
- No
- No answer

**18. What is your personal attitude toward:**

*(scale from 0 to 10; 0 = predominantly negative, 10 = predominantly positive)*

- Fertility treatment via ICSI and/or IVF
- Ovarian tissue cryopreservation

**19. What is your personal attitude toward measures that are not allowed or not fully established in Germany?**

*(scale from 0 to 10; 0 = predominantly negative, 10 = predominantly positive)*

- Oocyte donation
- Uterus transplantation
- Surrogacy
- Social freezing (oocyte cryopreservation without medical indication to allow future use)

**20. In your opinion, should the following methods be legalized in Germany?**

| **Method** | **Yes** | **Unsure** | **No** |
| --- | --- | --- | --- |
| Oocyte donation |  |  |  |
| Uterus transplantation |  |  |  |
| Surrogacy |  |  |  |

Participants were required to answer all questions to complete the questionnaire.
